# Racial disparities in conditional survival of patients with bladder cancer: a population-based study

**DOI:** 10.1186/s12894-023-01293-8

**Published:** 2023-07-18

**Authors:** Wei Liu, Jie Xiong, Honghao Wang, Shuo Li, Zhentao Lei, Lili Jiang, Jin Cao, Lin Yang, Hongfeng Guo, Qiang Gao, Shenghan Wang, Bao Zhang

**Affiliations:** 1 Department of Urology, Aerospace Center Hospital, Beijing, China; 2 Peking University Aerospace School of Clinical Medicine, Beijing, China; 3 Department of Urology, Zhongnan Hospital of Wuhan University, Wuhan, China

**Keywords:** Conditional survival, Definitive therapy, Racial disparity, Urinary bladder neoplasms

## Abstract

**Background:**

Traditional estimates can only provide static predictions of cancer outcomes and cannot assess the evolving effect of race on patient survival. This study aims to reveal the dynamic survival of patients with bladder cancer and to explore the evolving effect of race on patient prognosis.

**Methods:**

Using data from the Surveillance, Epidemiology, and End Results (SEER) registry, 99,590 white, 6,036 African American, and 4,685 Asian/Pacific Islander (API) patients with bladder cancer were identified. Conditional cancer-specific survival (CSS) rates, which could reflect the dynamic survival prediction of cancer patients, represented the primary outcomes, and were estimated by the Kaplan-Meier algorithm. The evolving effect of race on patient survival was evaluated by multivariable Cox regression in combination with conditional survival (CS) estimates.

**Results:**

The 5-year CSS for African American patients who had survived 1, 2, 3, 4, or 5 years after definitive therapy improved from the baseline calculation by + 5.8 (84.4%), + 9.5 (87.4%), + 12.8 (90.0%), + 14.4 (91.3%), and + 14.7% (91.5%), respectively. The increasing trend also held for overall white and API patients, and for all patient subsets when CS was calculated according to different levels of sex, age, and disease stage. African Americans, despite having the worst survival at baseline, could have CSS comparable to their white and API counterparts after 4 years of survivorship. In addition, the risk of death for African Americans tended to decrease with increasing survival, and the risk was no longer significantly different from that of whites after 4 years of survival.

**Conclusions:**

While having the worst initial predicted outcomes, African Americans may eventually achieve comparable survival to white and API patients given several years of survivorship. As patient survival increases, African American race may lose its role as an indicator of poorer prognosis.

**Supplementary Information:**

The online version contains supplementary material available at 10.1186/s12894-023-01293-8.

## Introduction

Bladder cancer represents the 10th most prevalent cancer worldwide [1]. In the United States, 81,400 new cases and 17,980 deaths from this disease are estimated to occur in 2020 [2]. Despite radical surgery, bladder cancer patients generally have a poor 5-year survival rate of 50 − 60% when the disease has progressed to an invasive stage [3]. In addition to known clinical and tumor-related factors, numerous studies have shown that racial and ethnic factors play a role in the survival of cancer patients [4–6]. Compared with white patients, African Americans tend to present with more advanced disease at diagnosis [7], are less likely to receive definitive treatment [8], and have poorer survival outcomes [5, 6].

Previous studies have reported racial and/or ethnic differences in survival of patients with bladder cancer [9–11], but many have been limited to giving traditional survival predictions by calculating the relative 5-yr or 10-yr overall survival/CSS rates. These statistics can only provide a static view and thus cannot reflect patients’ intrinsic survival that actually changes over time. On the contrary, CS calculations measure the probability that a cancer patient will survive some additional number of years, given the time the patient has survived [12, 13]. Therefore, as a practical tool, CS estimates can be used to predict the future survival of cancer patients at any given time, taking into account the time they have survived.

In this context, we relied on the CS algorithm to systematically reveal the dynamic survival characteristics of patients of different races and to explore the evolving effect of race on patient survival in a large cohort of bladder cancer. Based on the available evidence, we hypothesized that the outcome predictions for patients from different racial groups might evolve with longer survival time and that race might gradually lose its role as an independent indicator of prognosis over time.

## Materials and methods

### Data collection and study population

The data used in this study were collected from the SEER database of the National Cancer Institute program, which covers approximately 26% of the American population [14]. We used the SEER data to identify patients diagnosed with bladder cancer from 2004 to 2015. The year 2004 was selected as the initial year of this study given that several employed covariates were introduced in SEER in 2004 [15]. The disease diagnosis was determined according to the 10th revision of the International Classification of Disease for Oncology second edition (ICD-O-2) site codes (bladder: C67.0-67.9) and the ICD-O-3 histology codes (transitional cell carcinoma: 8120, 8122, 8130, and 8131). Patients with or without metastatic disease were included in the study to accommodate different study objectives. Cases were excluded if the age at diagnosis was less than 18 years; if the disease was reported through autopsy or death certificate; if bladder cancer was not the primary tumor; or if the demographic, clinical and follow-up data was incomplete. The detailed selection process of the cohort was shown in Supplementary Fig. 1.

### Covariates and outcome variables

We extracted patient-level demographics, including sex, race, age, marital status, and insurance status. Race was categorized as white, African American, API, and American Indian/Alaska Native. Each patient’s geographic region within the US (West, Northeast, South, or Midwest) and residence type (metropolitan vs. non-metropolitan) were also retrieved. Educational status (i.e., the percent of adults without a high school [HS] education) and median household income were determined at the county level, which was calculated by the SEER according to the Census American Community Survey 5-year data [15, 16]. Given the dynamic changes in census data, we ranked patients’ county-level education and median household income by quartiles rather than by specific numbers according to the year of diagnosis and the corresponding census data. Thus, for educational status, patients from the 1st quartile represented the most educated population, whereas those from the 4th quartile were classified as the least educated.

We also abstracted treatment-related variables (type of surgery, lymph node dissection status, radio- and/or chemotherapy utilization status) and pathologic variables (tumor stage, nodal stage, metastatic status, tumor grade, and lymphovascular invasion). Considering the update of the database, and to ensure consistent staging criteria for the included patients, we relied on the 6th edition of the American Joint Committee on Cancer (AJCC) Staging Manual for bladder cancer [17] to extract patient staging information.

For outcome variables, we investigated whether it differed across racial groups regarding the diagnostic yield of advanced disease and the incidence of receipt of appropriate definitive therapy. In this study, inoperable locally advanced (T4b and any N; or any T and N2–3) or metastatic (M1) bladder cancer was considered as advanced disease [18, 19]. Appropriate definitive therapy was defined as surgery and/or radiotherapy for patients with bladder cancer and was indicated for patients with non-advanced disease for whom staging and treatment information was available. Relative CSS rates and conditional 5-yr CSS rates were considered the primary outcomes of interest. CSS was defined as the interval from primary treatment to death from bladder cancer.

### Statistical analyses

Baseline patient characteristics were compared using the analysis of variance (ANOVA) for continuous variables and the χ^2^ test for categorical measures. The multivariable logistic regression was employed to determine the race(s) associated with increased odds of presenting with advanced disease after adjusting for demographic variables (sex, age, year of diagnosis, marital status, insurance status, geographic region, residence type, education, and median household income). The same method was used to examine whether the incidence of receiving definitive therapy differed by race after adjusting for the demographic factors and tumor-related variables (tumor stage, nodal stage, and tumor grade) in the regression model. Odds ratios (ORs) and 95% confidence intervals (CIs) were calculated using statistical tools.

For survival statistics, we relied on the Kaplan-Meier method to estimate CSS in the overall population and in patients with different stages of disease. CS estimates were calculated using the multiplicative law of probability. Specifically, CS refers to the probability of surviving *x* additional years (CS_*x*_) given *y* years of accumulated survival [CS_*x*_ = S_(*x*+*y*)_)/S_(*y)*_] [20–22]. In the current study, conditional CSS was first quantified using the Kaplan-Meier method for different racial groups. Then, conditional 5-yr CSS calculations were performed according to patient age, sex, and disease stage, respectively. Stratified analyses were considered and comparisons between different groups were performed using the log-rank test. Multivariable Cox regression models were used to assess the dynamic effect of race on cancer-specific mortality which took into account the time patients had survived after adjusting for demographic factors, tumor-related variables, and the use of definitive therapy. Hazard ratios (HRs) and 95% CIs were estimated using statistical tools. All statistical tests were two-tailed, and the significance level was set at *P* < 0.05. Analyses were performed with GraphPad Prism v.5.0 (GraphPad Software Inc) and SPSS v.25.0 (IBM Corp).

## Results

### Patient characteristics

Baseline characteristics of the included patients are shown in Supplementary Table 1. Overall, we identified 110,311 patients who met the study inclusion criteria: 99,590 were white, 6,036 were African American, and 4,685 were API (American Indians/Alaska Natives were not included because of the small number of cases). The median follow-up was 85 months. Compared with white and API patients, African Americans in the cohort were younger, had a higher percentage of female enrollees, and were more likely to be unmarried (comparisons among the three race arms, all *P* < 0.001, Supplementary Table 1). African Americans generally had less insurance coverage, lower levels of education, and lower household incomes (comparisons among the three race arms, all *P* < 0.001, Supplementary Table 1). Regarding clinical variables, African Americans were more likely to present with higher tumor grades and disease stages (Supplementary Table 1 and Table 1), and had a lower utilization of definitive therapy compared to their white and API counterparts (Supplementary Table 1 and Table 1).

**Table 1:** Associations between race and outcomes of presenting with advanced disease, and use of definitive therapy

Races	Advanced disease at diagnosis†				Definitive therapy‡		
	OR§	95% CI	*P*-value		OR¶	95% CI	*P*-value
White	Ref.				Ref.		
African American	1.64	1.46–1.83	< 0.001		0.80	0.71–0.91	0.001
API	1.05	0.90–1.23	0.541		0.95	0.82–1.11	0.543

Abbreviations: API, Asian/Pacific Islander; CI, confidence interval; OR, odds ratio

†This study defined inoperable locally advanced (T4b and any N; or any T and N2–3) or metastatic (M1) bladder cancer as advanced disease

‡Excluded patients with advanced disease

§OR adjusted for demographic variables (sex, age, year of diagnosis, marital status, insurance status, geographic region, residence type, education, and median household income)

¶OR adjusted for demographic variables and tumor-related factors (tumor grade, tumor stage, and nodal stage)

### Racial disparities in traditional survival

**Fig. 1:**
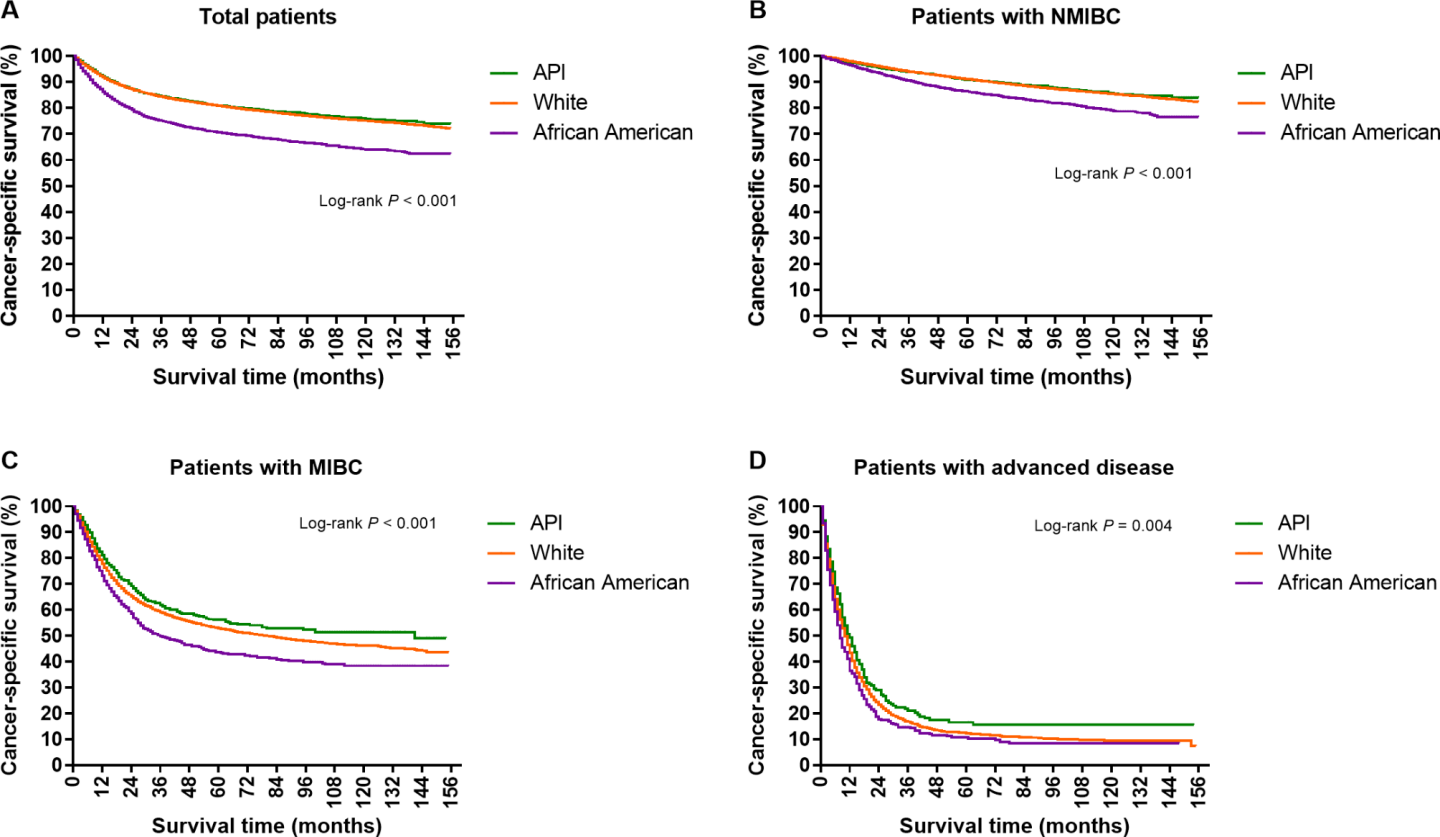
Racial disparities in baseline cancer-specific survival among patients with bladder cancer. Baseline cancer-specific survival estimates for overall patients **(A)**, patients with non-muscle invasive disease **(B)**, patients with muscle invasive disease **(C)**, and patients with advanced disease **(D)**. API, Asian/Pacific Islander; MIBC, muscle invasive bladder cancer; NMIBC, non-muscle invasive bladder cancer

Figure 1 shows the survival curves of bladder cancer patients with or without definitive therapy. Overall, significant differences were observed in CSS among the API, white, and African American populations (*P* < 0.001; Fig. 1A), whether they had non-muscle invasive bladder cancer (NMIBC) (*P* < 0.001; Fig. 1B), muscle invasive bladder cancer (MIBC) (*P* < 0.001; Fig. 1C), or advanced disease (*P* = 0.004; Fig. 1D). Considering the 5-yr CSS, the survival probabilities for API, white, and African American patients with NMIBC disease were 90.8%, 91.1%, and 86.4%, respectively (Fig. 1B). For patients diagnosed with MIBC, the probabilities decreased to 56.1%, 52.8%, and 43.5% for the three racial groups, respectively (Fig. 1C). After that, patients with advanced disease had the worst survival with 5-yr CSS of 16.6%, 12.4%, and 10.8% for API, white, and African American patients, respectively (Fig. 1D).

### Racial disparities in conditional survival

**Fig. 2:**
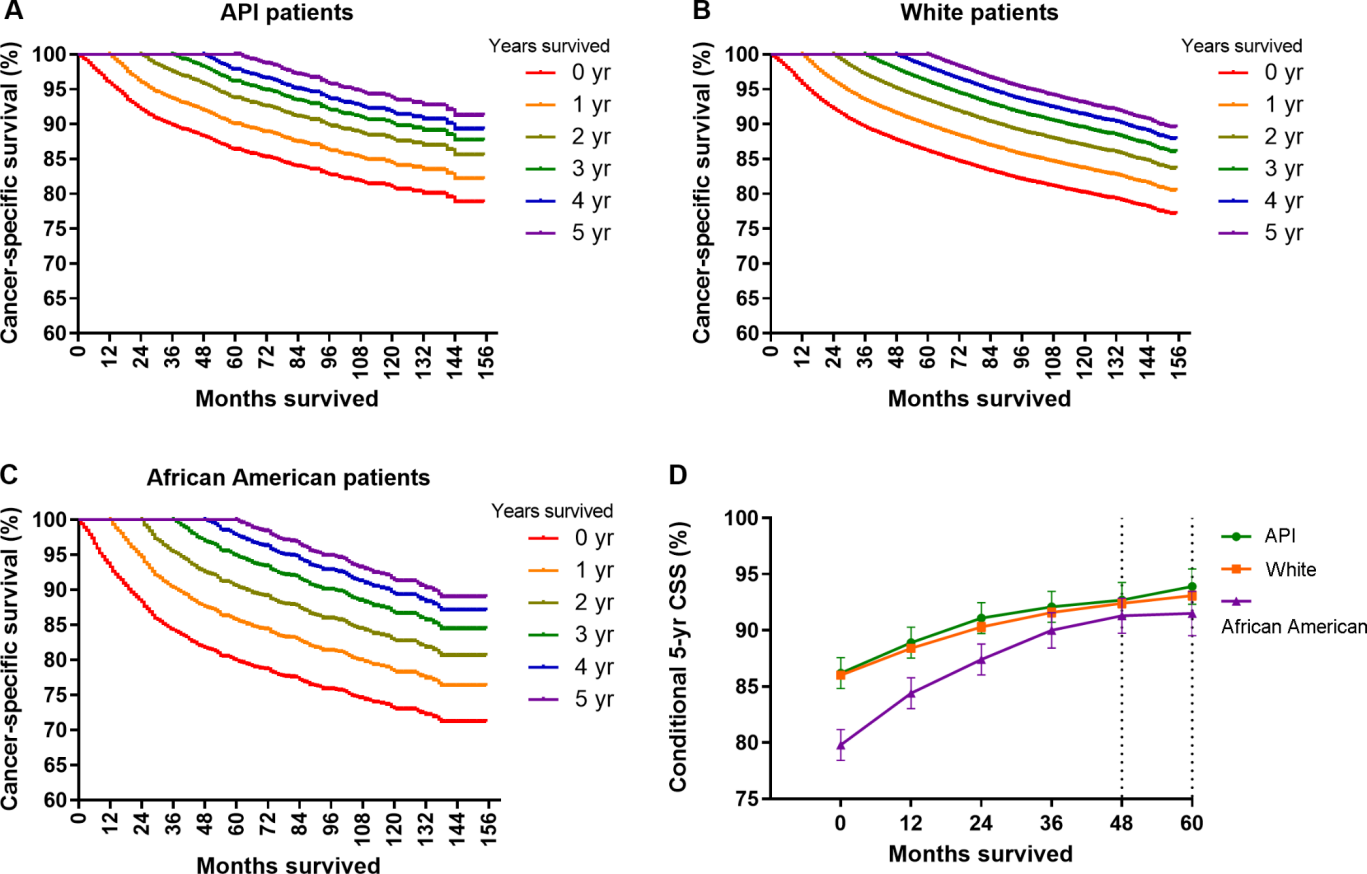
Conditional survival curves for bladder cancer patients according to survived time since definitive treatment. Conditional cancer-specific survival curves were plotted for Asian/Pacific Islander (API) patients **(A)**, white patients **(B)**, and African American patients **(C)**. **(D)** shows the overall changing trend of 5-yr cancer-specific survival in three racial groups after accounting for the time patient survived. Time points with vertical dashed lines (Fig. 2D) indicate no statistically significant differences in patient survival among the three racial groups, otherwise there is a significant difference

The CS estimates demonstrated an improving pattern for bladder cancer patients since definitive treatment, regardless of their race categories (Fig. 2A-C). Specifically, for API patients, the baseline 5-yr CSS rate was 86.2%. Given 1-, 2-, 3-, 4-, and 5-yr survivorship, the survival probabilities increased to 88.9%, 91.1%, 92.1%, 92.7%, and 93.9%, respectively (Fig. 2D). Similarly, the 5-yr CSS for African American patients who had survived 1, 2, 3, 4, or 5 years after definitive therapy improved from the baseline calculation by + 5.8 (84.4%), + 9.5 (87.4%), + 12.8 (90.0%), + 14.4 (91.3%), and + 14.7% (91.5%), respectively (Fig. 2D). Interestingly, despite having the worst CS in the first few years after definitive therapy, African American individuals could have CSS comparable to their white and API counterparts after 4-yr survivorship (5-yr CSS at year 4: 92.7%, 92.4%, and 91.3% for API, white, and African American patients, respectively, *P* = 0.210; Fig. 2D).

**Fig. 3:**
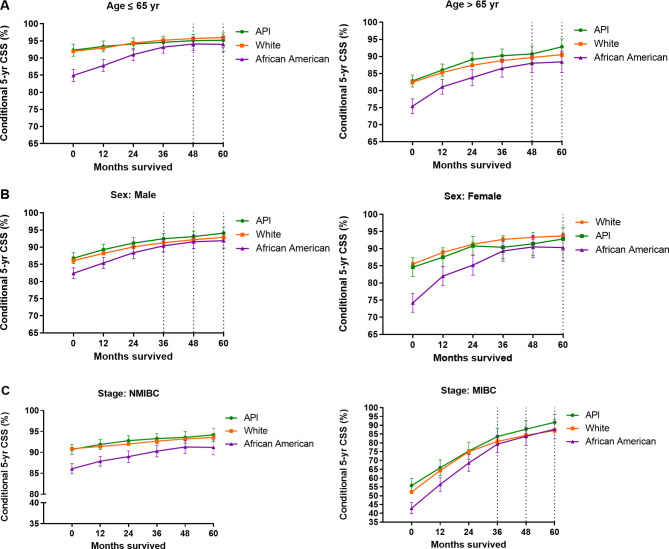
Racial disparities in conditional 5-yr cancer-specific survival among bladder cancer patients stratified by different levels of covariates. The Kaplan-Meier method was employed to calculate conditional 5-yr cancer-specific survival rates for patients of different age **(A)**, sex **(B)**, and disease stage **(C)** stratified by race, according to the number of years the patient survived. Error bars represent 95% confidence intervals (CIs). Time points with vertical dashed lines indicate no statistically significant difference in patient survival among the three racial groups, otherwise there is a significant difference. API, Asian/Pacific Islander; MIBC, muscle invasive bladder cancer; NMIBC, non-muscle invasive bladder cancer

Figure 3 shows racial disparities in conditional 5-yr CSS according to the levels of different covariates, age, sex, and disease stage. Overall, elderly patients (age > 65 year), female individuals, and patients with MIBC disease had worse CSS at baseline (time zero) compared to their younger, male, and NMIBC counterparts, respectively. As expected, African American patients had the worst CSS at baseline, regardless of their strata according to age, sex, and disease stage. However, the survival pattern could change as patients survived longer. When analyzed by age (Fig. 3A), there were no significant differences in CS among the three racial groups after 4 years of survival. African American males had an increase in 5-yr CSS from 82.4% at baseline to 90.4% at 3 year since definitive therapy, and thereafter maintained survival comparable to white and API patients (Fig. 3B). For African American females, a remarkable 21.7% improvement of CSS was recorded from baseline to 5 year after definitive treatment (Fig. 3B). When stratified by disease stage (Fig. 3C), patients with MIBC ultimately achieved a greater improvement in CSS compared to patients with early-stage disease, despite worse survival at baseline. Specifically, given 3-yr survivorship, the 5-yr CSS rate for African Americans with MIBC increased to 79.3%, which was comparable to the 83.6% and 80.8% for API and white patients, respectively (Fig. 3C). For NMIBC patients, however, the CS rates underwent a slow climb, and in African Americans with more improved survival, the 5-yr CSS increased slightly from 86.1% at baseline to 91.2% at 5 year, resulting in the persistence of racial differences in patient survival. The aforementioned improving trends were similarly recorded when CS was calculated according to tumor grades (Supplementary Fig. 2) and AJCC integrated stages (Supplementary Fig. 3).

### Cox regression analyses

**Fig. 4:**
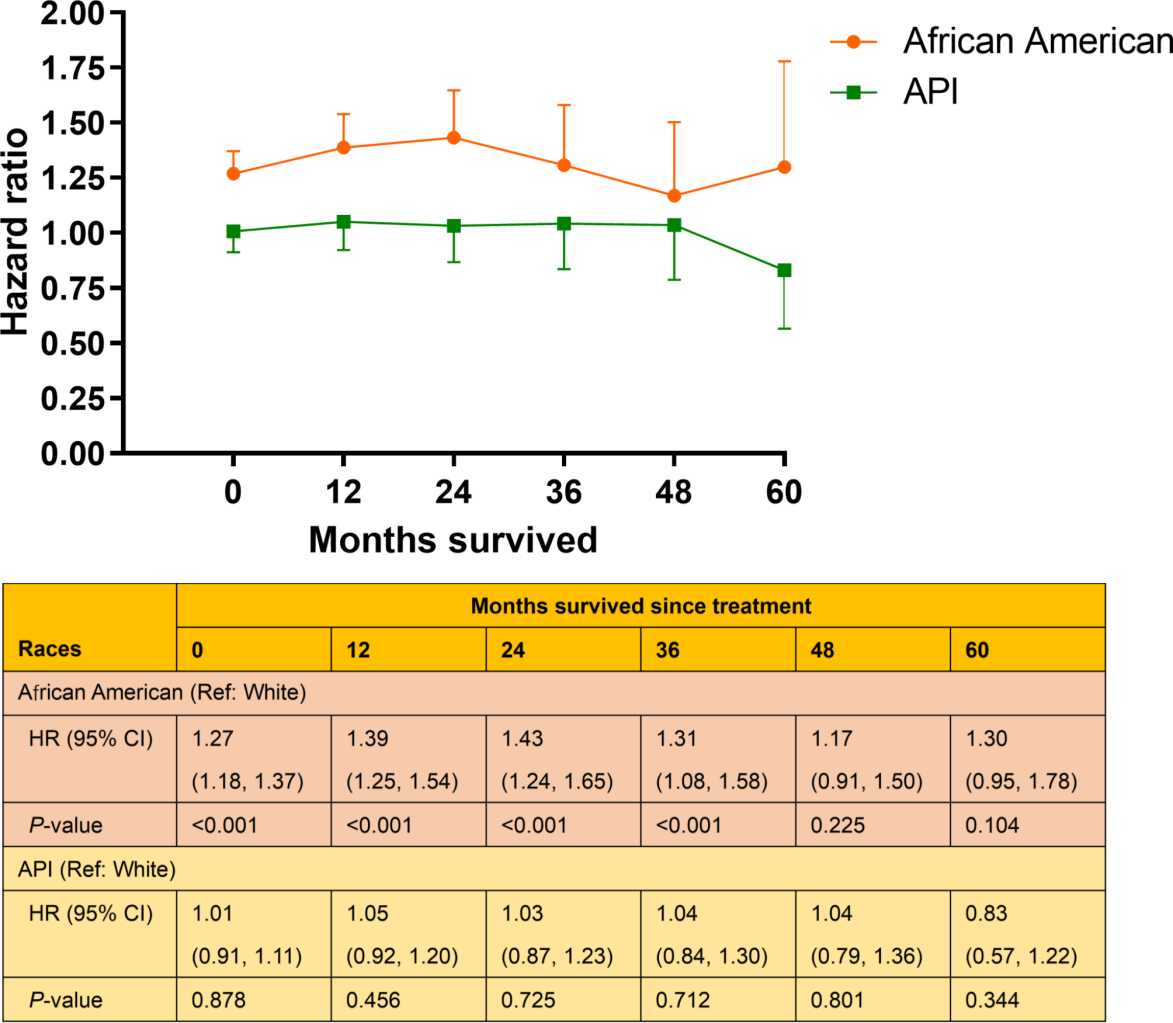
Changes in proportional hazard ratios in multivariable Cox regression models for each racial population compared with whites. Error bars represent 95% confidence intervals (CIs). API, Asian/Pacific Islander; HR, hazard ratio

In univariable Cox regression analyses, API and white patients had a similar risk of cancer-specific mortality at baseline (HR, 0.96; 95% CI, 0.87–1.05; data not shown), whereas African Americans had a 47.7% higher risk of mortality than whites (HR, 1.48; 95% CI, 1.37–1.59; data not shown). To determine whether the aforementioned racial differences in CS could be explained by other confounding factors, we performed a multivariable Cox regression analysis to simultaneously control for other covariates, including demographic factors, tumor-related variables, and receipt of definitive therapy. The results showed that African Americans had a significantly higher risk of death than their white counterparts until survival to 4 year after treatment, whereas no significant differences were detected between API and white patients from baseline to 5-yr survivorship (Fig. 4).

## Discussion

Racial differences in cancer survival have long been a topic. Although whites have twice the incidence of bladder cancer as African Americans, the latter have the opposite mortality rate [23]. Previous studies have documented racial disparities in bladder cancer survival, but many have been limited to estimates by calculating static survival rates and have failed to evaluate the evolving effect of race on patient prognosis. To more realistically characterize the survival of bladder cancer patients, we designed the current study to investigate the dynamic survival of white, African American, and API patients using CS methods. The results mainly showed the advantages for whites and APIs with better baseline survival. Although African American patients have the worst initial predicted outcomes, they can ultimately achieve comparable survival to white and API patients after several years of survivorship. As patient survival increases, African American race may lose its role as an indicator of poorer prognosis.

Traditionally, relative 5-yr or 10-yr survival rates serve as a calculator to give survival predictions for cancer patients. Despite this convention, these statistics can only provide a static view on patient prognosis. In contrast, CS estimates can more accurately reflect the changing risk of death for cancer patients by calculating the probabilities of survival for any additional years, taking into account the cumulative survival time [24]. To date, the CS algorithm has been employed to depict patient survival in melanoma [25], gastrointestinal cancers [20, 26], malignant hematologic diseases [27], and other primary malignancies [12, 28]. In practice, CS estimates can be useful for both patients and physicians. For patients, CS estimates can provide individualized survival predictions for the next few years at any point since disease diagnosis or initiation of treatment [14]. And for physicians, CS estimates can be used to adjust the monitoring regimen by considering the cumulative survival and the risk of death over time [14].

In our study, we initially wondered how patient survival evolved in different racial groups and whether race could persistently act as an independent prognostic factor as patients survive longer. Using the CS method, we found that the CSS estimates for bladder cancer patients demonstrated an improving pattern since definitive treatment in all three racial groups. Interestingly, despite having the worst initial prediction, African Americans could have a prognosis comparable to whites and API patients after 4 years of survival (Fig. 2D). This similar trend still held for all subsets when CS was calculated in different strata, including age, sex, and disease stage (except for NMIBC patients).

When stratified by age, patients older than 65 year, though having a poorer survival at baseline, ultimately achieved a greater improvement in CS compared with their younger counterparts (age ≤ 65 year). Furthermore, the records showed that after 4 years of survival, there was no longer a significant difference in prognosis among the three racial populations, either in patients > 65 year or ≤ 65 year. Wang et al. documented a similar trend of CS improvements in different racial groups according to patient age in lung cancer [24]. These results may suggest that as survival time increases, the elderly African Americans may have a comparable long-term prognosis to elderly API and white patients. For bladder cancer, female patients generally had worse survival than male individuals [29, 30]. Despite this challenge, with longer survival time, the females in our study could achieve a more remarkable survival improvement than the males (Fig. 3B). More importantly, even with the dual burden of adverse factors (being African American and being female), African American females could unexpectedly achieve comparable 5-yr CSS to whites and APIs after 5 years of survivorship. Therefore, African American females may expect comparable survival to white and/or male patients once they survive the critical few years after definitive treatment. When analyzed by disease stage, patients with MIBC had a greater improvement in CS estimates compared to patients with NMIBC. The reason for this may be a better survival in NMIBC patients at baseline and there leaves little room for further improvement. However, for MIBC patients, the baseline survival is conversely worse, and thus their long-term outcomes may improve significantly with prolonged survivorship.

When considering the dynamic effect of race on patient survival, a decreasing trend in mortality was observed among African American patients and surprisingly, the African American-white discrepancy could disappear after 4 years of survival. In other words, as patient survival increases, race may gradually lose its role as a prognostic indicator.

In addition to the aforementioned significance, our observations may have important implications for prognostic evaluation and surveillance planning. Using CS estimates, we can provide individualized dynamic survival predictions, rather than static calculations, for patients from different racial groups according to their clinical characteristics (age, sex, and socioeconomic status), tumor grade, disease stage, and treatment modality. With this facility, it also allows patients to quantify their survival improvements in long-term follow-up. Based on the current data, we would additionally recommend the inclusion of race as a prognostic factor in patient counseling and follow-up planning. Specifically, in the first few years after treatment, African Americans may indicate a worse prognosis, thus necessitating enhanced surveillance of this population and adoption of quality healthcare. Once out of the critical 4 years of survivorship, as demonstrated by the results, patients will no longer experience racial differences in survival and may expect a better outcome. Thus in the following years, examinations with longer intervals can be considered. In addition to guiding surveillance, this information may also strengthen patients’ confidence to overcome the disease and encourage them to adopt more aggressive treatments.

Despite these strengths, the current study had several limitations that warrant mention. First, due to limitations in data availability, the study was unable to examine the effects of other confounders such as lifestyle (e.g., smoking and alcohol consumption), occupational exposures, and biological factors on patient survival. The non-inclusion of these factors hindered a more in-depth interpretation of the African American-white differences in survival. Second, the participants in this study were collected from the US population. Whether the recorded findings can be extrapolated to patients in other countries or regions requires further investigation. Nonetheless, to our knowledge, it remains a rare study that comprehensively analyzes racial disparities in dynamic survival in a large bladder cancer cohort. The current records also have important implications for patient counseling and follow-up planning.

## Conclusions

African American patients, while having the worst initial prediction, may eventually achieve comparable survival to white and API patients after a few years of survivorship. As patient survival increases, African American race may lose its role as an indicator of poorer prognosis. The current records may have important implications for patient counseling and development of follow-up plans.

## Electronic supplementary material

Below is the link to the electronic supplementary material.

**Supplementary Table 1:** Baseline characteristics of included 110,311 patients with bladder cancer. **Supplementary Fig. 1:** Flowchart of patient selection of the bladder cancer cohort. **Supplementary Fig. 2:** Conditional 5-yr cancer-specific survival according to different levels of tumor grades. **Supplementary Fig. 3:** Conditional 5-yr cancer-specific survival according to different levels of AJCC integrated stages.

## Data Availability

The data of this study are available from the corresponding author upon reasonable request.

## Abbreviations

AJCC: American Joint Committee on Cancer
ANOVA: Analysis of variance
API: Asian/Pacific Islander
CI: Confidence interval
CS: Conditional survival
CSS: Cancer-specific survival
HR: Hazard ratio
ICD-O: International Classification of Disease for Oncology
IQR: Interquartile range
MIBC: Muscle invasive bladder cancer
NMIBC: Non-muscle invasive bladder cancer
OR: Odds ratio
SD: Standard deviation
SEER: Surveillance, Epidemiology, and End Results
