# Supplementary material for: Racial disparities in conditional survival of patients with bladder cancer: a population-based study
Source: BMC Urol. 2023 Jul 18;23:122. doi: 10.1186/s12894-023-01293-8 (PMC10354880; doi:10.1186/s12894-023-01293-8)
Supplement: Supplementary file 1 — Supplementary Table 1: Baseline characteristics of included 110,311 patients with bladder cancer. Supplementary Fig. 1: Flowchart of patient selection of the bladder cancer cohort. Supplementary Fig. 2: Conditional 5-yr cancer-specific survival according to different levels of tumor grades. Supplementary Fig. 3: Conditional 5-yr cancer-specific survival according to different levels of AJCC integrated stages. [file 12894_2023_1293_MOESM1_ESM.doc]

**Supplementary data**

**Supplementary Table 1** Baseline characteristics of included 110,311 patients with bladder cancer

| Characteristics | Overall (n = 110,311), No. (%) | White (n = 99,590), No. (%) | African American (n = 6,036), No. (%) | API (n = 4,685), No. (%) | *P*-value |
| --- | --- | --- | --- | --- | --- |
| Year of diagnosis |  |  |  |  |  |
| 2004 – 2007 | 37,912 (34.4) | 34,459 (34.6) | 1,971 (32.7) | 1,482 (31.6) | < 0.001 |
| 2008 – 2011 | 35,249 (31.9) | 31,927 (32.1) | 1,855 (30.7) | 1,467 (31.3) |  |
| 2012 – 2015 | 37,150 (33.7) | 33,204 (33.3) | 2,210 (36.6) | 1,736 (37.1) |  |
| Sex |  |  |  |  |  |
| Male | 84,012 (76.2) | 76,397 (76.7) | 4,068 (67.4) | 3,547 (75.7) | < 0.001 |
| Female | 26,299 (23.8) | 23,193 (23.3) | 1,968 (32.6) | 1,138 (24.3) |  |
| Age at diagnosis (yr) |  |  |  |  |  |
| Mean ± SD | 69.8 ± 12.1 | 70.0 ± 12.1 | 67.5 ± 12.4 | 70.3 ± 12.6 | < 0.001 |
| ≤ 65 yr | 38,895 (35.3) | 34,694 (34.8) | 2,624 (43.5) | 1,577 (33.7) | < 0.001 |
| > 65 yr | 71,416 (64.7) | 64,896 (65.2) | 3,412 (56.5) | 3,108 (66.3) |  |
| Marital status |  |  |  |  |  |
| Married | 66,079 (59.9) | 60,453 (60.7) | 2,481 (41.1) | 3,145 (67.1) | < 0.001 |
| Unmarried | 36,887 (33.4) | 32,439 (32.6) | 3,171 (52.5) | 1,277 (27.3) |  |
| Unknown | 7,345 (6.7) | 6,698 (6.7) | 384 (6.4) | 263 (5.6) |  |
| Region |  |  |  |  |  |
| West | 51,209 (46.4) | 46,186 (46.4) | 1,849 (30.6) | 3,174 (67.7) | < 0.001 |
| Northeast | 22,202 (20.1) | 20,883 (21.0) | 987 (16.4) | 332 (7.1) |  |
| South | 26,570 (24.1) | 22,955 (23.0) | 2,504 (41.5) | 1,111 (23.7) |  |
| Midwest | 10,330 (9.4) | 9,566 (9.6) | 696 (11.5) | 68 (1.5) |  |

Supplementary Table 1 continued

| Characteristics | Overall (n = 110,311), No. (%) | White (n = 99,590), No. (%) | African American (n = 6,036), No. (%) | API (n = 4,685), No. (%) | *P*-value |
| --- | --- | --- | --- | --- | --- |
| Urban-rural residence |  |  |  |  |  |
| Metropolitan area | 95,635 (86.7) | 85,639 (86.0) | 5,522 (91.5) | 4,474 (95.5) | < 0.001 |
| Non-metropolitan area | 14,676 (13.3) | 13,951 (14.0) | 514 (8.5) | 211 (4.5) |  |
| Median household income |  |  |  |  |  |
| 1st quartile | 27,349 (24.8) | 25,001 (25.1) | 2,174 (36.0) | 174 (3.7) | < 0.001 |
| 2nd quartile | 27,935 (25.3) | 24,563 (24.7) | 1,951 (32.3) | 1,421 (30.3) |  |
| 3rd quartile | 27,549 (25) | 25,757 (25.9) | 956 (15.8) | 836 (17.8) |  |
| 4th quartile | 27,478 (24.9) | 24,269 (24.4) | 955 (15.8) | 2,254 (48.1) |  |
| Insurance status |  |  |  |  |  |
| Insured | 77,245 (70.0) | 69,615 (69.9) | 4,219 (69.9) | 3,411 (72.8) | < 0.001 |
| Uninsured | 1,570 (1.4) | 1,315 (1.3) | 185 (3.1) | 70 (1.5) |  |
| Unknown | 31,496 (28.6) | 28,660 (28.8) | 1,632 (27.0) | 1,204 (25.7) |  |
| % without HS education |  |  |  |  |  |
| 1st quartile | 27,669 (25.1) | 25,543 (25.6) | 905 (15.0) | 1,221 (26.1) | < 0.001 |
| 2nd quartile | 28,949 (26.2) | 26,247 (26.4) | 1,406 (23.3) | 1,296 (27.7) |  |
| 3rd quartile | 26,218 (23.8) | 23,353 (23.4) | 1,968 (32.6) | 897 (19.1) |  |
| 4th quartile | 27,475 (24.9) | 24,447 (24.5) | 1,757 (29.1) | 1,271 (27.1) |  |
| Tumour grade |  |  |  |  |  |
| Well differentiated | 16,694 (15.1) | 15,346 (15.4) | 859 (14.2) | 489 (10.4) | < 0.001 |
| Moderately differentiated | 32,854 (29.8) | 29,972 (30.1) | 1,502 (24.9) | 1,380 (29.5) |  |
| Poorly differentiated | 22,355 (20.3) | 20,069 (20.2) | 1,428 (23.7) | 858 (18.3) |  |
| Undifferentiated | 38,408 (34.8) | 34,203 (34.3) | 2,247 (37.2) | 1,958 (41.8) |  |
|  |  |  |  |  |  |

Supplementary Table 1 continued

| Characteristics | Overall (n = 110,311), No. (%) | White (n = 99,590), No. (%) | African American (n = 6,036), No. (%) | API (n = 4,685), No. (%) | *P*-value |
| --- | --- | --- | --- | --- | --- |
| AJCC stage |  |  |  |  |  |
| 0 | 57,502 (52.1) | 52,644 (52.9) | 2,564 (42.5) | 2,294 (49.0) | < 0.001 |
| Ⅰ | 27,232 (24.7) | 24,401 (24.5) | 1,557 (25.8) | 1,274 (27.2) |  |
| Ⅱ | 13,670 (12.4) | 12,193 (12.2) | 904 (15.0) | 573 (12.2) |  |
| Ⅲ | 4,623 (4.2) | 4,058 (4.1) | 338 (5.6) | 227 (4.8) |  |
| Ⅳ | 7,284 (6.6) | 6,294 (6.3) | 673 (11.1) | 317 (6.8) |  |
| Definitive treatment |  |  |  |  |  |
| Yes | 76,947 (69.8) | 69,540 (69.8) | 3,950 (65.4) | 3,457 (73.8) | < 0.001 |
| No | 9,844 (8.9) | 8,704 (8.7) | 728 (12.1) | 412 (8.8) |  |
| NA/Unknown | 23,520 (21.3) | 21,346 (21.4) | 1,358 (22.5) | 816 (17.4) |  |
| Follow-up |  |  |  |  |  |
| Median months (IQR) | 85 (44, 120) | 86 (45, 120) | 79 (41, 118) | 78 (37, 116) |  |
| Overall deaths | 45,595 (41.3) | 41,060 (41.2) | 2,875 (47.6) | 1,660 (35.4) | < 0.001 |
| Cancer-specific deaths | 21,662 (19.6) | 19,115 (19.2) | 1,696 (28.1) | 851 (18.2) | < 0.001 |

Abbreviations: AJCC, American Joint Committee on Cancer; API, Asian/Pacific Islander; HS, high school; IQR, interquartile range;

NA, not applicable; SD, standard deviation. Percentages may not add up to 100 due to rounding.

**Supplementary figures**

**
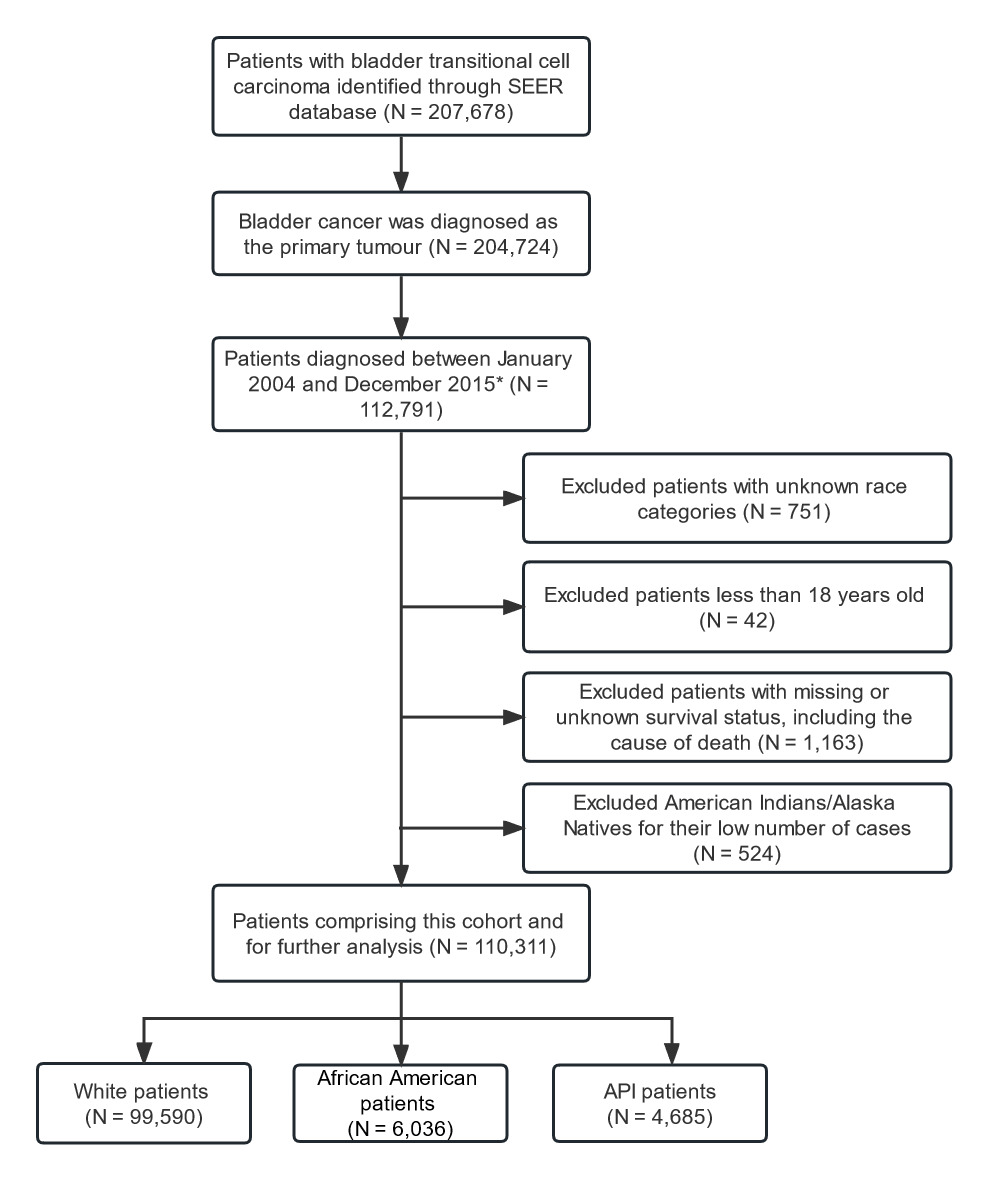
**

**Supplementary Fig. 1.** Flowchart of patient selection of the bladder cancer cohort.

^*^The year 2004 was selected as the initial year of this study given that several employed covariates were introduced in SEER database in 2004. The period 2004 – 2015 was selected to ensure consistent staging criteria (AJCC 6th edition) for the included patients. API, Asian/Pacific Islander.


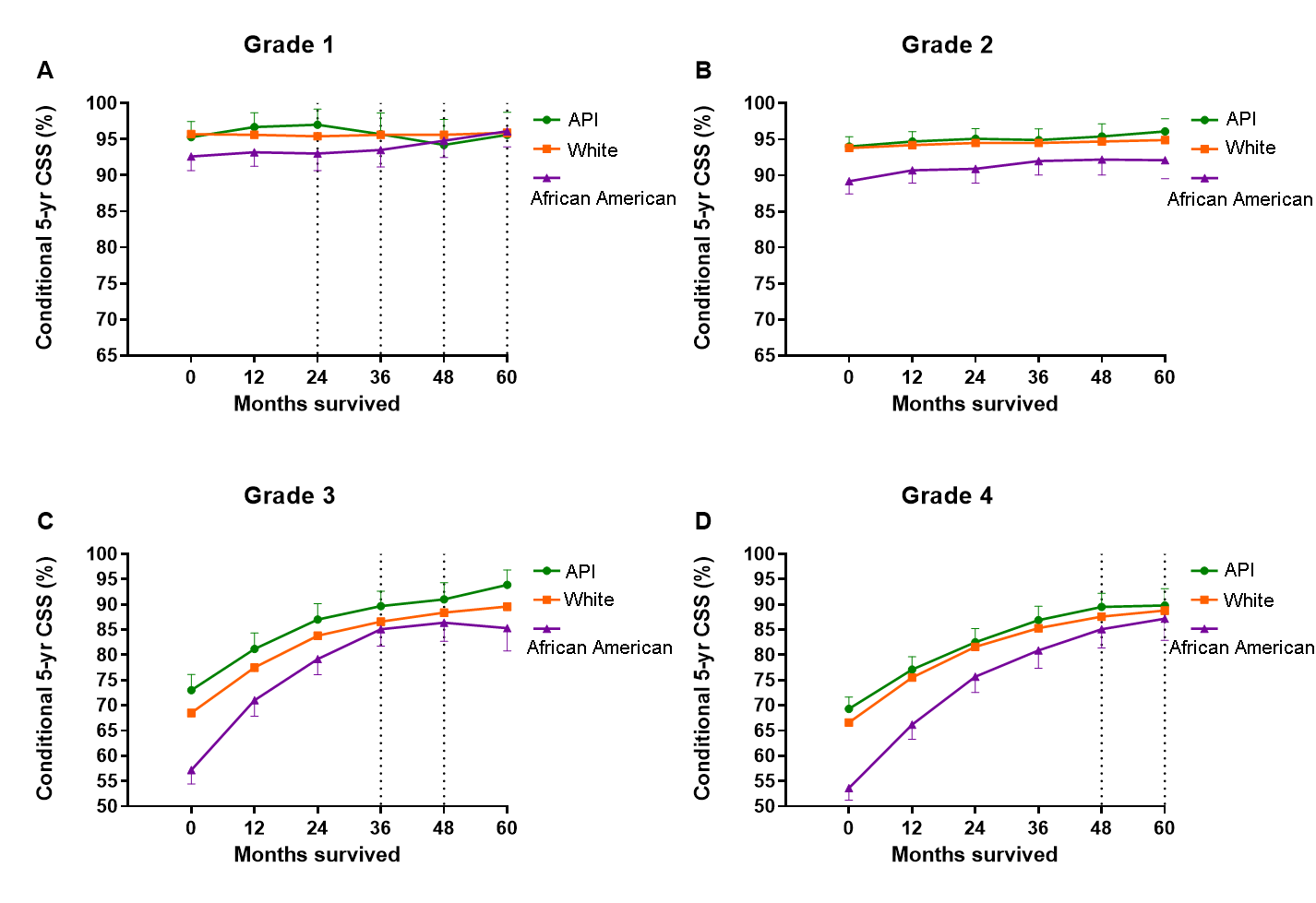


**Supplementary Fig 2.** Conditional 5-yr cancer-specific survival according to different levels of tumor grades. The changing trend in conditional 5-yr cancer-specific survival (CSS) across racial groups was depicted for patients diagnosed with well differentiated (Grade 1; A), moderately differentiated (Grade 2; B), poorly differentiated (Grade 3; C), and undifferentiated (Grade 4; D) bladder cancer, according to the number of years patient survived. Time points with vertical dashed lines indicate no statistically significant differences in patient survival among the three racial groups, otherwise there is a significant difference. API, Asian/Pacific Islander.


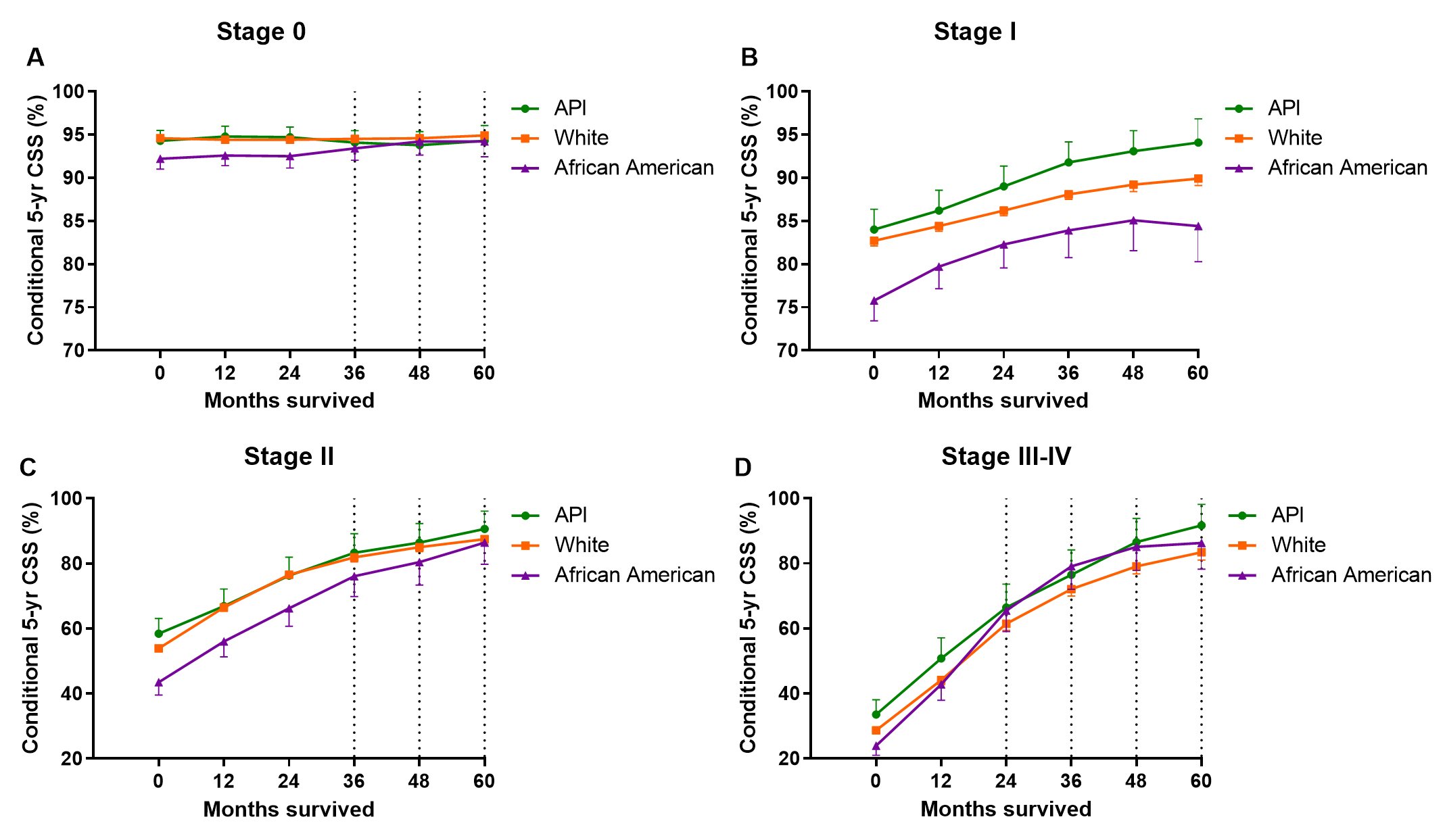


**Supplementary Fig 3.** Conditional 5-yr cancer-specific survival according to different levels of AJCC integrated stages. The conditional 5-yr cancer-specific survival (CSS) rates were respectively calculated for patients with stage 0 (A), Stage Ⅰ (B), stage Ⅱ (C), and stage Ⅲ - Ⅳ (D) disease taking the survived time into account. Time points with vertical dashed lines indicate no statistically significant differences in patient survival among the three racial groups, otherwise there is a significant difference. AJCC, American Joint Committee on Cancer; API, Asian/Pacific Islander.
